# High‐Entropy Materials for Prospective Biomedical Applications: Challenges and Opportunities

**DOI:** 10.1002/advs.202406521

**Published:** 2024-09-09

**Authors:** Ling Chang, Haochuan Jing, Chao Liu, Chuantian Qiu, Xiang Ling

**Affiliations:** ^1^ International Collaborative Laboratory of 2D Materials for Optoelectronics Science and Technology of Ministry of Education Institute of Microscale Optoeletronics Shenzhen University Shenzhen 518060 China; ^2^ Department of Nuclear Medicine Yunnan Cancer Hospital and The Third Affiliated Hospital of Kunming Medical University Kunming 650000 China; ^3^ ZJU‐Hangzhou Global Scientific and Technological Innovation Center Zhejiang University Hangzhou 311215 China

**Keywords:** biomedical, cocktail effect, high‐entropy materials, multi‐element components, multi‐functional materials

## Abstract

With their unique structural characteristics, customizable chemical composition, and adjustable functional characteristics, high‐entropy materials (HEMs) have triggered a wide range of interdisciplinary research, especially in the biomedical field. In this paper, the basic concept, core properties, and preparation methods of HEMs are first summarized, and then the application and development of HEMs in the field of biomedical are briefly described. Subsequently, based on the diverse and comprehensive properties of HEMs and a few reported cases, the possible application scenarios of HEMs in biological fields such as biosensors, antibacterial materials, therapeutics, bioimaging, and tissue engineering are prospectively predicted and discussed. Finally, their potential advantages and major challenges is summarized, which may provide useful guidance and principles for researchers to develop and optimize novel HEMs.

## Graphical abstract

Description of a multi‐layer doughnut diagram from the inside out. First, HEMs are divided into HENMs and bulk HEMs according to different scales. Then, the preparation methods of HENMs are divided into three categories: top‐down, bottom‐up and topochemical transformation. Finally, its applications in five fields such as biosensor, antibacterial, antitumor, bioimaging and tissue engineering are introduced.

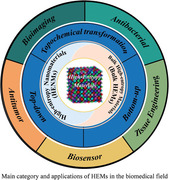

## 1 Introduction

Major changes in various advanced technologies have often relied on the development and application of new materials. Different materials have traditionally been combined to achieve better performance or produce entirely new features. Mixing materials at the atomic level can result in highly disordered, multi‐component, high‐entropy systems, including single‐phase solid solutions with simple crystal structures,^[^ ^1^ ^]^ lattice distortions, and properties surpassing those of the constituent materials.^[^ ^2^ ^]^ Although the concept of high‐entropy alloys was proposed as early as 2004,^[^ ^3^ ^]^ it was not until 2015 that the central role of entropy in driving the transition to a homogeneous single‐phase system was first confirmed,^[^ ^4^ ^]^ and the entropy‐stable oxide family was introduced. As a result, the scientific community has focused on the clear verification of entropy stability. Subsequently, many types of high‐entropy materials (HEMs) have been explored, including alloys,^[^ ^5^ ^]^ oxides,^[^ ^6^ ^]^ sulfide,^[^ ^7^ ^]^ carbides,^[^ ^8^ ^]^ nitrides,^[^ ^9^ ^]^ and borides.^[^ ^10^ ^]^

In 2016, the synthesis of multi‐element nanoparticles was achieved through the use of restricted nanoreactors.^[^ ^11^ ^]^ Specifically, after a series of high‐entropy nanomaterials (HENMs) were obtained by researchers in 2018 through a non‐equilibrium thermal shock method,^[^ ^12^ ^]^ HENMs quickly became the research focus of functional nanomaterials.^[^ ^13^ ^]^ Compared with bulk HEMs, HENMs offer an attractive material platform for a wider range of application scenarios. The obvious lattice distortions in HENMs can reduce the overall system energy, further promoting the activation and transport of active substances, resulting in the expanded study of fields such as energy technology,^[^ ^14^ ^]^ electrocatalysis,^[^ ^15^ ^]^ thermal catalysis,^[^ ^16^ ^]^ thermoelectric conversion,^[^ ^17^ ^]^ and plastic recycling.^[^ ^18^ ^]^ In recent years, the controlled synthesis of various low‐dimensional HENMs, including 0D,^[^ ^12^, ^19^ ^]^ 1D,^[^ ^5^, ^20^ ^]^ and 2D^[^ ^21^ ^]^ HENMs, has also been rapidly developed. These HENMs with specific microstructures not only provide a research basis for theoretical calculation but also greatly enrich the application range of HEMs. With increasing demand for new materials in the biomedical field, the nanoscale engineering of bulk HEMs can accelerate their applications.

HENMs are highly applicable in the biomedical field due to their unique properties,^[^ ^22^ ^]^ exceeding the performance of a single‐element composition and offering customizable functional characteristics. For example, HENMs have demonstrated excellent performance in the field of biosensing by finetuning their structure and elemental composition.^[^ ^23^ ^]^ Based on semiconductor properties and uniquely designed ion dissolution properties, HENMs can effectively inhibit the growth and reproduction of bacteria,^[^ ^5^, ^24^ ^]^ providing a new powerful tool for antibacterial therapy. In addition to realizing photothermal and photodynamic therapy using their excellent light absorption ability,^[^ ^25^ ^]^ HENMs can introduce special reactivity such as the Fenton reaction through specific metals (i.e., Fe, Cu, and Mn),^[^ ^26^ ^]^ providing new means for tumor treatment. In addition, the incorporation of some special elements (i.e., Pt, Pd, W) has allowed for these materials to be widely used in biological imaging and disease diagnosis, providing doctors with more accurate disease localization and treatment methods.^[^ ^27^ ^]^ Notably, the design of HENMs has not only focused on functionality but also fully considered biosafety. Through purposeful design, these materials may exhibit high hardness and wear resistance, significantly reducing the potential toxicity of heavy metal ions in the body during biodegradation when used as implants.^[^ ^28^ ^]^ Compared with other nanomaterials, the high‐entropy characteristics of HENMs surfaces may be more conducive to cell adhesion and proliferation,^[^ ^29^ ^]^ improving the medical effect. In addition, the multi‐functional element‐based interface interaction of HENMs may be realized by modifying the material surface.^[^ ^30^ ^]^ These advantages provide HENMs with great application potential in the biomedical field.

**Figure 1:**
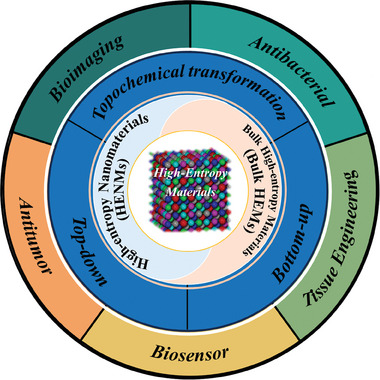
An overview of the main category and applications of HEMs in the biomedical field.

Although the application of HEMs in the biological field remains at an early stage with few reported literature reviews, their unique characteristics and potential application prospects have attracted significant attention. To fully understand and promote the development of HEMs in the biological field, in this review, we discussed the following aspects in depth (**Figure** **1**). First, we outlined two theoretical concepts, four comprehensive effects, and three preparation strategies of HEMs, laying a theoretical foundation for the subsequent discussion of application in the biological field. Second, we introduced the development of HEMs in the biological field. From their initial exploration stage to their rapid development today, we demonstrated their ever‐expanding application scope and potential. Next, we focused on the unique characteristics of HEMs, combined with a few existing application cases, and prospectively predicted and explored their application in biosensing, antibacterial materials, therapeutic, bioimaging, and tissue engineering. Finally, we discussed the upcoming opportunities and challenges in this emerging and highly promising field. We further explored how to overcome existing technical difficulties and obstacles to provide strong support for the development of HEMs in the biological field. This review aims to serve as a valuable reference and inspiration for the development of HEMs in the biological field, to accelerate progress and innovation in this field.

## 2 Theoretical Definition and Structural Properties

HEMs can be considered the optimization of performance by introducing the atoms of various other elements to modulate the valence state and electron density of the target atom.^[^ ^31^ ^]^ This modulation strategy can provide HEMs with unique physical and chemical properties, resulting in significant application potential in many fields, including the biological field. Although HEMs have been extensively studied, their definition has remained controversial, with two definitions currently widely adopted. The initial definition, introduced in 2004, was centered on ingredient necessities and commonly encompassed a material made up of five or more elements in approximately equivalent molar or equal molar concentrations. Each constituent element within this material was required to possess an atomic percentage ranging from 5% to 35%.^[^ ^32^ ^]^ This definition implicitly encompasses HEMs where the atomic percentage of the additive element is minimal or even falls below 5%. Alternatively, this definition can be stated as:

(1) $$n_{major}\geq 5.5\;\mathrm{at}.\%\leq c_{i}\leq 35\;\mathrm{at}.\%$$

(2) $$n_{minor}\geq 0,c_{i}\leq 5\;\mathrm{at}.\%$$

where n*_major_* and n*_minor_* indicate the quantity of primary and secondary elements, respectively. Furthermore, c*_i_* and c*_j_* refer to the respective atomic percentages of the primary and secondary elements. Under this definition, the HEMs system may comprise an equilibrium of elements in an equal molar ratio, a blend of various non‐equivalent molar components, or an assembly of multiple sub‐molar elements. The core principle underlying this definition lies in leveraging high mixing entropy to facilitate the creation of solid solution materials. Alternatively, the second definition frames HEMs as materials that exhibit a configurational entropy exceeding 1.61R in a randomized solution state, emphasizing the entropic perspective:

(3) $$\Delta S_{conf}=\;-R[c_{1}\ln c_{1}+c_{2}\ln c_{2}+\cdot \cdot \cdot +c_{n}\ln c_{n}$$

(4) $$\;\;=\;-R\sum_{i\;=\;1}^{n}c_{i}\;\ln c_{i}$$

where R is the gas constant.

According to the extreme value theorem, when *c_1_* = *c_2_* = ··· = *c_n_*, the entropy of the system will reach the maximum Rln*n*. For ideal HEMs, the closer the component content, and the greater the mixing entropy. Formula 3 can be derived from the regular solution model, when the energy is high enough, any node position in the crystal structure can be randomly occupied by atoms of different elements (Figure 3B). The random miscible state of the material is similar to that of an ideal solution; thus, the mixing entropy of the material can be determined using Formula 4. Based on this calculation, materials can be categorized into four distinct groups: 1) ultra‐pure materials, also referred to as 0 entropy materials or high‐purity materials, exhibiting a theoretical entropy approaching 0; 2) low‐entropy materials, characterized by a configurational entropy of S*_conf_* ≤ 0.69R, with one or two primary elements; 3) medium‐entropy materials, having a configurational entropy within the range of 0.69R ≤ S*_conf_* ≤ 1.61R and containing two to four main elements; 4) high‐entropy materials, defined by a configurational entropy of S*_conf_* ≥ 1.61R and comprising at least five elements. Notably, compared with solid solution HEMs with single‐phase crystal structures such as face‐centered cubic (fcc), body‐centered cubic (bcc), and hexagonal close‐packed (hcp), HEMs with dual‐phases or multi‐phases not only exhibit excellent performance in enhancing physical characteristics such as the strength and plasticity of materials^[^ ^32^ ^]^ but have also shown great potential in expanding application functionality.

As mentioned above, it can be divided into block high‐entropy materials and HENMs by the distinction of material size, and the main characteristics of this classification are reflected in the echo of application scenarios in the biomedical field. In addition, the components and properties of the elements involved can be classified into: high entropy alloy (HEA), high entropy oxide (HEO), high entropy sulfide (HES), high entropy carbide (HEC) and high entropy nitride (HEN). Several representative products related to biomedical applications are summarized in **Table** **1**. Although this classification is more detailed, we will still refer to bulk HEMs and HENMs separately later in the article, which will help them to reflect the critical factor of size in their respective application scenarios

**Table 1:** Various HEMs with different morphology and application scenarios.

Categories	Materials	Morphology	Application	Ref.
HEA	PtPdRuRhIr	Ultrafine‐nanoparticles	Antitumor	[25]
HEA	FeCuAgCeGd	Ultrafine‐nanoparticles	Biosensor	[23a]
HEA	FeNiTiCrMn	Nanoparticle	Antibacterial	[33]
HEA	TiZrNbTaMo	Bulk	Bone implant	[34]
HEA	Ta‐Ti‐Nb‐Zr	Powder	3D bio‐scaffold	[35]
HEA	Fe‐Co‐Ni‐Cu	Nanoparticle	Antitumor	[36]
HEA	PtPdMnCoFe	Nanochain‐like internetworks	Biosensor	[37]
HEA	TiZrHfTa	Bulk	Bone implant	[38]
HEA	PtPdRhCuM (M = Mn, Co or Ni)	Nanotube	Biosensor	[39]
HEA	HfNbTaTiZr	Bulk	Bioimplant	[40]
HEA	PtRhMoCoFe	Nanodendrites	Biosensor	[5c]
HEO	NiCoCrCuFe(O)	Nanoparticle	Biosensor	[6b]
HEO	NbTaTiVZr(O)	Film	Bone implant	[41]
HES	(ZnCdFeMnCu)_x_S	Hollow nanocube	Biosensor	[23b]
HES	(ZnCdCuAgCo)_x_S	Nanorod	Biosensor	[7b]
HEC	TiVCrMoAlC_3_	Nanosheet	Antibacterial	[21c]
HEC	(TiZrNbTaHf)C	Film	Bone implant	[42]
HEC	TiVNiMoC	Nanosheet	Antibacterial and skin tissue repair	[43]
HEC	TiVCrMoC_3_	Nanosheet	Antibacterial	[44]
HEN	(TiZrNbHfTa)N	Film	Bone implants	[8c]

The multiple elements that can be modulated and the diversification of preparation methods endow HEMs with a high‐entropy effect, sluggish diffusion effect, severe lattice distortion effect, and cocktail effect, which makes them have better performance. (Figure 3A).^[^ ^45^ ^]^ For example, adjusting electronic interaction between different elements can affect the adsorption–interface interaction, assisting in the development of HENM catalysts with high stability and energy advantages.^[^ ^46^ ^]^ Lattice distortions and surface strain, as well as the chemical diversity of adsorption sites, can improve the catalytic activity, selectivity, and durability of HENMs.^[^ ^47^ ^]^

Based on its unique multi‐element composition, HEMs is significantly different from traditional monadic or binary material systems, thus demonstrating many extraordinary properties.^[^ ^32^, ^45^ ^]^ We describe the four effects and their implications for biomedical applications as follows:

1) High entropy effect, as a signature feature of HEMs, is of self‐evident importance. This effect, by inhibiting the formation of stoichiometric compounds that are usually highly ordered and brittle, in turn promotes the formation of solid solution phases (SSPS), thus giving the material more excellent and diverse physicochemical properties.^[^ ^1^ ^]^ They are widely used in implant and repair materials, anti‐bacterial and anti‐tumor therapy, and multi‐modal biological imaging.^[^ ^23^, ^48^ ^]^ By fine‐tuning the element composition ratio, not only achieve a high degree of biocompatibility to the human body, but also give these materials a variety of functional properties.
2) In HEMs, the adjacent solute atoms cause significant lattice distortion due to size differences and bond asymmetry, and the atoms deviate from their ideal positions. This lattice distortion not only profoundly changes the structural response of the material under stress deformation, but also directly enhances the hardness and strength of the material, making it show higher stability and durability when subjected to external loads.^[^ ^1^ ^]^ The severe lattice distortion effect in HEMs significantly enhances its interaction with human tissues by improving the mechanical properties and surface properties of the material. This distortion not only promotes the adhesion and growth of cells on the surface of the material,^[^ ^49^ ^]^ but also improves the corrosion resistance and wear resistance of the material.
3) The slow diffusion effect is due to the complexity of the moving path of atoms or vacancies in the lattice, which inhibits the overall dynamic process and slows down the phase transition speed.^[^ ^45b^ ^]^ This effect not only affects the microstructure evolution of materials, but also provides a possibility for fine regulation of material properties. It effectively slows down the corrosion and wear rate of the alloy in the human body and prolongs the service life of the implant.^[^ ^41^ ^]^ Thus, the slow diffusion effect also shows a unique advantage in the biomedical field.
4) In addition, the cocktail effect further reveals the complexity and diversity of HEMs performance optimization. The synergies of multiple main elements not only superimpose their independent effects, but also have indirect effects on the microstructure of the material through complex interaction mechanisms.^[^ ^45a^ ^]^ This combined effect makes HEMs excellent in many aspects such as strength, toughness, fatigue resistance, creep resistance, thermal and electrical conductivity. In this way, the optimization and coordination of material properties are realized by precisely regulating the types and proportions of elements in the HEMs. This enables HEMs to accurately respond to external fields (such as biosensing signals, tumor microenvironments, etc.),^[^ ^5c^ ^]^ meeting specific medical needs such as biosensing, tumor therapy, and tissue repair.

These characteristics have resulted in a wealth of changes in physical and chemical properties, including good thermal stability,^[^ ^50^ ^]^ excellent corrosion resistance,^[^ ^51^ ^]^ high hardness,^[^ ^52^ ^]^ as well as unique physical (electro‐optical^[^ ^53^ ^]^ and magnetic^[^ ^54^ ^]^) and chemical (catalytic,^[^ ^5^, ^16^, ^55^ ^]^ semiconductor,^[^ ^56^ ^]^ and biological behavior^[^ ^57^ ^]^) properties. In addition, the phase, microstructure, and component elements of biocompatibility have shown significant roles in the biomedical field of HEMs.^[^ ^48^ ^]^ High entropy materials have shown wide application potential in many fields such as biomedicine and engineering technology due to their unique properties. These performance advantages not only enhance the practicality of the material, but also lay a solid foundation for future development in many fields.

## 3 Synthetic Methods for HENMs

The field of HEMs is clearly divided into two categories: bulk HEMs and HENMs, and their preparation strategies are quite different. Although the bulk HEMs have multiple components, according to the existing research and application experience, the preparation methods of bulk HEMs are very similar.^[^ ^1^ ^]^ Such as the fusion‐cast process, physical vapor deposition, powder metallurgy, melt spinning, etc.^[^ ^32^ ^]^ We will not repeat these traditional preparation methods, we focus on the preparation of HEMs. There are three main preparation methods of HENMs: bottom‐up method, top‐down method, and topochemical transformation method (**Table** 2).

### 3.1 Bottom‐Up Method

The bottom‐up method refers to the gradual construction of nanoscale materials or structures from basic units such as atoms, molecules or clusters through chemical reactions or physical processes. Its advantage is that it can prepare nanostructures with uniform chemical composition and few defects, and it can precisely control the size, morphology and properties of nanomaterials by regulating the reaction conditions.^[^ ^58^ ^]^ Because of the particularity of the composition of HEMs, it has many characteristics in the bottom‐up method. It is usually a combination of several methods such as high thermal pyrolysis, template assisted, sol‐gel assisted, hydrothermal synthesis and high temperature calcination.

#### 3.1.1 High Thermal Pyrolysis

**Figure 2:**
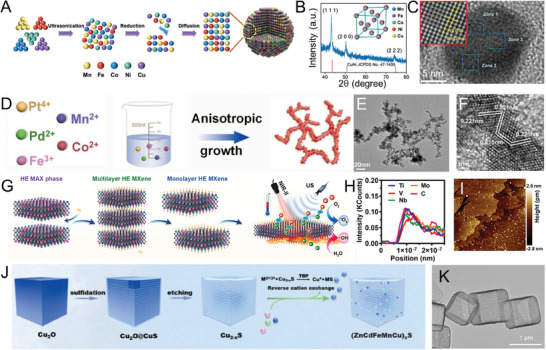
A) Schematic illustration showing the formation process of the HEA NPs. B) XRD pattern and crystal structure (inset). C) HRTEM image. The inset diagram shows the atomic arrangement.^[^ ^5a^ ^]^ Copyright 2023, John Wiley and Sons. D) Schematic diagram for construction of the PtPdMnCoFe HEAINN. E) and F) Low‐ and high‐resolution TEM images of the PtPdMnCoFe HEAINN.^[^ ^37^ ^]^ Copyright 2024, Elsevier. G) Schematic illustration of the main synthesis procedure of oxygen vacancy and wide interlayer gap TiVNiMnC Mxene, and NIR‐II‐mediated ROS generation for enhancing the SDT efficacy. H) The location of the EDS line scan of the monolayer TVNMC Mxene. I) AFM micrograph of the monolayer TiVNiMnC MXene. Copyright 2024, Elsevier.^[^ ^43^ ^]^ J) schematic diagram of (ZnCdFeMnCu)_x_S reaction to synthesize HENMs based on cation exchange. K) TEM images of (ZnCdFeMnCu)_x_S nanocubes.^[^ ^23b^ ^]^ Copyright 2024, Elsevier.

The high thermal pyrolysis method has the advantage of preparing nanoparticles with specific size and controllable morphology. Therefore, HENMs can be obtained by pyrolysis and reduction of a variety of metal salts.^[^ ^5^, ^20^, ^59^ ^]^ With acetylacetone salts of Mn, Fe, Co, Ni, and Cu as metal precursors and glucose as reducing agent, MnFeCoNiCu transition metal high entropy nanozymes (HEzymes) were prepared by using hexadecyltrimethyl ammonium bromide (CTAB) and oleoamine (OAm) as solvents for the first time and structural inducers with unique surface atomic configurations and obvious D‐orbital coupling characteristics of different metal components^[^ ^5a^ ^]^ (Figure **2A–C**). Its excellent peroxidase (POD‐like) activity is employed for biosensing and antibacterial applications. Similarly, homogeneous PtRhMoCoFe high entropy alloy dendrites (HEANDs) were prepared using glucose and oleoamine as co‐reducing agents.^[^ ^5c^ ^]^ An electrochemical label free biosensor constructed by HEANDs was used to realize the ultrasensitive determination of biomarker cTnI. A series of magnetically rich Fe‐Co‐Ni‐Cu high‐entropy magnetic nanoparticles (HEMNPs) were successfully prepared by the same high thermal decomposition technology. Among them, Fe_70_Co_14_Ni_14_Cu_2_, which has an absolute advantage in iron content, showed distinct ferromagnetic characteristics.^[^ ^36^ ^]^ This sample, when applied as a magnetic fluid (MNP concentration: 2 mg ml^−1^), demonstrated an efficient ability to rapidly raise the temperature to a level sufficient to kill cancer cells. This finding not only validates the effectiveness of this synthetic strategy, but also indicates its potential to extend to the development of high performance, high entropy magnetic nanomaterials in the field of magnetothermal therapy (MT). High entropy nanomaterials prepared by high thermal pyrolysis have significant advantages, but their biocompatibility is affected by oily ligands. If the ligands on the surface of the material can be effectively removed, it can not only improve its applicability in the biological field, but also significantly enhance its detection sensitivity and many properties, such as stability and reaction rate, so as to promote its application in the biomedical field.

#### 3.1.2 Template Assisted

The soft and hard template assisted is suitable for constructing HENMs with specified structure, which has potential guiding value^[^ ^19^, ^60^ ^]^ for implementing different application directions. PtPdRhRuCu MMNs obtained by using block copolymer micelles (PEO‐b‐PMMA) as soft template have uniformly distributed mesoporous (≈ 23 nm). A large number of exposed mesoporous make them have high efficiency ion migration and high density of high entropy alloy sites, and the high entropy alloy sites are thought to have low energy barrier due to multi‐element synergies.^[^ ^19^ ^]^ The high entropy alloying of various metal elements was achieved by using KIT‐6 as the hard limited domain template during calcination at high temperature, and a series of high entropy nanospheres with ordered pores were obtained by etching the hard template by HF.^[^ ^60b^ ^]^ Self‐supported PtPdMnCoFe high‐entropy alloy with nanochain‐like internetworks (PtPdMnCoFe HEAINN) It is thought to have been obtained by a one‐pot co‐reduction method using H_2_ gas produced during the decomposition of NaBH_4_ as a template^[^ ^37^ ^]^ (Figure 2D–F). The high‐entropy structure can effectively reduce H_2_O_2_ and is used as a signal amplifier of a labeling free electrochemical amperometric immunosensor for the determination of neuronspecific enolase (NSE). HENMs of Cu_2_Zn_1_Al_0.5_Ce_5_Zr_0.5_O_x_ oxide were synthesized using 2D micelles formed by PVP as templates.^[^ ^21b^ ^]^ The use of template assisted is helpful for the synthesis of HENMs with specified structures, which can accurately control the size, shape, structure and properties of nanomaterials. This is achieved by selecting suitable template materials and structures and conducting the synthesis of nanomaterials under the guidance of the template. This precise control makes the template method a significant advantage when preparing nanomaterials with specific performance requirements.

#### 3.1.3 Sol‐Gel Assisted

Sol‐gel assisted is a colloid with uniform composition obtained by direct mutual diffusion of chemical elements, which has significant advantages in accurately ensuring the proportion of each component of the material for the preparation of multi‐component materials with variable composition proportions. Therefore, stabilizing the precursor of multiple elements with the aid of gel, and then calcining and decomposition of the precursor is also a common method to obtain HENMs.^[^ ^15^, ^25^, ^61^ ^]^ Under the stability of PVP, the metal supramolecular polymer precursors were prepared by crosslinking of organometals with organic ligand molecules. The US‐HEANPs obtained by calcination had excellent POD‐like activity, and could catalyze endogenous H_2_O_2_ to generate toxic hydroxyl radical (·OH).^[^ ^25^ ^]^ In addition, US‐HEANPs has a high photothermal conversion effect under 808 nm infrared light (NIR) irradiation. Through the interaction of enzyme‐like catalytic free radical generation activity and efficient photothermal action, the prepared US‐HEANPs can effectively eliminate tumor cells. F127 as a gelling agent can also be used to stabilize the cations of various metal salts to achieve the preparation of HENMs.^[^ ^6c^ ^]^ In addition, a very similar synthesis method is to mix a variety of metal elements out of solution by forming complexes in the wet chemical synthesis stage, and then calcination at high temperature to obtain HENMs.^[^ ^23^, ^62^ ^]^ It can be seen that both sol‐gel and complex formation methods are to obtain a precursor of a variety of elements fully mixed in advance, which is an effective way to anchor atoms to prevent movement, and then obtain HENMs in the process of high temperature calcination. Special elements can be added according to the needs to achieve its function.

Finally, the luminol‐dissolved O_2_ (DO) electrochemiluminescence (ECL) sensing is achieved by hydrothermal reaction combined with calcination to obtain high entropy oxides.^[^ ^6b^ ^]^ It is also the synthesis of spherical mesoporous HEOs (e.g., Ni‐Co‐Cr‐Fe‐Mn oxide) by hydrothermal reaction combined with sol‐gel method and calcination, which is a nano platform for efficient detection of DNA.^[^ ^6c^ ^]^ Usually, the preparation process of hydrothermal and solvothermal is similar, and it can also be used for the preparation of new nanomaterials.^[^ ^63^ ^]^ In addition, to prevent phase separation, researchers have developed a variety of ultra‐fast synthesis techniques including carbothermal shock method,^[^ ^12^ ^]^ fast moving bed^[^ ^64^ ^]^ and flame spray pyrolysis^[^ ^6^, ^65^ ^]^ are a variety of kinetic nonequilibrium approaches. This provides more means for the research and development of high performance and HENMs, and also greatly enriched the library of HENMs.

### 3.2 Top‐Down Method

The top‐down method is to start from the macroscopic scale bulk material and cut, grind or strip it into nanoscale materials or structures by physical or chemical means. This method usually involves the action of external energies such as mechanical forces, ion beams, and lasers. The advantage of top‐down method is that existing macroscopic materials can be used as raw materials, but defects or impurities may be introduced in the preparation process, and it is difficult to accurately control the size and morphology of nanomaterials. Common top‐down methods include liquid phase exfoliation, mechanical ball milling, laser ablation and so on.

#### 3.2.1 Liquid‐Phase Exfoliation

The liquid‐phase exfoliation is to destroy the interaction between layers through a series of chemical reactions and physical reactions, and finally achieve the stripping of layered materials. The most typical example is the stripping of high entropy MAX phase block material with layered structure. Chemical liquid‐phase exfoliation of HE MXene is usually accompanied by selective etching of Al by MAX and LiF.^[^ ^21^, ^43^ ^]^ Due to the combination of various elements, the etched HEMXene (TiVNbMoC_3_) is highly likely to possess semiconductor properties, at which time its photothermal conversion ability and ultrasonic free radical generation properties can be utilized to achieve antibacterial and promote tissue repair under the intervention of light and ultrasound^[^ ^43^ ^]^ (Figure 2G–I). The heterojunction formed between HEMXene and CDs after HE 2D material stripping can realize broad spectrum antibacterial effect under near infrared light.^[^ ^44^ ^]^ However, the liquid phase stripping process will make the 2D HE material size smaller, generally less than 5 µm. In addition, the strong force may also cause local defects of HE 2D materials, affecting their properties. In addition, the 2D HEMXene material obtained after liquid phase exfoliation has a large specific surface area, which may adsorb solvent molecules, surfactants and debris involved in the stripping process, resulting in sample pollution. In addition, liquid phase stripping method is often used to achieve the preparation of lamellar HENMs with weak interaction force with the help of ultrasonic wave, ball milling and ion intercalation.^[^ ^21a^ ^]^ This method often uses harmful HF as the etching agent, and the yield is low, so it is not suitable for mass synthesis.

#### 3.2.2 Mechanical Ball Milling

The mechanical ball milling method can make use of the continuous friction, collision and shear between the ball and the material to gradually crush, refine and mix the material evenly. The bulk HEMs can be reduced to nanometer size or a variety of single‐component ball milling into a mixed powder with multiple components. This method does not require complex chemical reagents or high temperature and high pressure conditions to achieve a variety of materials crushing, alloying and composite material preparation.^[^ ^66^ ^]^ Mechanical ball milling method has been widely used in material science, metallurgy, chemical industry and other fields because of its simple operation, low cost and wide range of application. In the process of ball milling, the particle size distribution, phase composition and microstructure of the product can be adjusted by precisely controlling the parameters of ball milling time, speed, type and size of the ball. For example, mechanical ball grinding into NiFeCrCoCu HEA nanoparticles ‐graphene composite properties are affected.^[^ ^67^ ^]^ The monolayer HEMXene obtained by ball milling combined with selective etching have good NIR‐II region photothermal effect and extremely high biocatalytic activity.^[^ ^21c^ ^]^ It can be used as a nano agent to treat bacterial keratitis (BK) and subcutaneous abscess infection caused by methicillin‐resistant *Staphylococcus aureus* (MRSA) by the endo‐oxidase‐mimicking activity of IR‐II. In the process of producing CuCoNiFeMn HEA by high‐energy ball milling at room temperature,^[^ ^68^ ^]^ compared with the short HEBM process, the five elements of HENMs can be evenly distributed in the long HEBM process, and there is no obvious aggregation.

#### 3.2.3 Laser Ablation

Laser ablation is the production of nanoscale films or nanoparticles by the ablation of the corresponding material precursor by a focused (usually pulsed) laser beam, which typically takes only 5 nanoseconds per pulse to obtain the corresponding nanoparticle. The ultra‐fast ablation process ensures that different metal elements combine with each other, overcomes the influence of various elements in terms of thermodynamic solubility,^[^ ^58^ ^]^ and can effectively achieve the surface treatment and chemical transformation of the material.^[^ ^69^ ^]^ Since the laser pulse limits the energy to the desired micro‐region, the laser scanning ablation method enables the high‐entropy material nanoparticles to be loaded onto a variety of substrates, including heat‐sensitive substrates.^[^ ^58^ ^]^ The stability, size distribution and morphology of target colloidal nanoparticles can be effectively controlled by precisely adjusting laser parameters such as laser power, laser repetition rate, ablation rate and specific ion effect of solvent. Pulsed laser ablation of liquid medium can prepare stable colloidal HfNbTaTiZr refractory high entropy alloy NPs without any stabilizer, and has high photothermal conversion efficiency under 640 nm irradiation, which provides conditions for photothermal antibacterial and photothermal tumor therapy.^[^ ^70^ ^]^ HENMs are prepared by direct ablation without stabilizer, the process is simple and efficient, and cumbersome additive steps are eliminated. The obtained product has high purity and no interference from impurities, which provides great convenience and reliability for the subsequent application, and ensures the accuracy of the experimental results and the stability of the material properties. This fast and controllable synthesis method enables HENMs to have a large number of repeatable properties. CoCrFeNiMn HENMs with five metal components uniformly distributed were prepared by ultrashort pulse laser ablation.^[^ ^71^ ^]^ Although the sizes of the synthesized HENMs vary the diameters of most NPs can be stabilized at ≈2.8 nm. The laser ablation technique can obtain stable synthesized HENMs by precisely adjusting multiple parameters, which enables the performance of the target material to be reproduced within a certain tolerance range. This technical method ensures the purity of HENMs in the preparation process, and then shows excellent stability advantages in use, providing a reliable material basis for various application scenarios.

Samples with different Cu content (FeNiTiCrMnCux) were synthesized by non‐equilibrium arc discharge plasma method. The samples showed excellent light absorption and photothermal conversion performance in the whole solar spectrum (250‐2500 nm).^[^ ^33^ ^]^ FeNiTiCrMnCu_1.0_ HEA‐NPs (400 µg mL^−1^) also be proved the significant antibacterial performance under the irradiation of solar energy (1 sun), and this excellent improvement was related to the synergistic effect of copper ion release and photothermal damage. High entropy alloy nanoparticles with FCC single‐phase structure were obtained by DC arc plasma discharge. Micron high entropy alloy prepared with ball milling as the precursor, the maximum number of elements mixed reached 7 and the distribution of elements was uniform.^[^ ^66a^ ^]^ The results show that the material exhibits 96% absorption properties at the spectrum of 250 to 2500 nm. Compared with the laser ablation technique, the fine control ability of the arc discharge method in the experimental process is limited, especially in the temperature control there are significant challenges, so the performance and quality reproducibility of the synthesized material is poor.

### 3.3 Topochemical Transformation Method

Topochemical transformation is also a simple and convenient method for the preparation of HENMs, which is usually through cation exchange, dealloying, selective REDOX and sulfide nitridation methods to achieve precise changes in the specified elements. The core of this method is that the structure or form of the product is obtained through topochemical changes in the precursor of the reactant (i.e., the intermolecular or interatomic connection and spatial arrangement). This is also an effective strategy to obtain HENMs with specific structure and function after the preparation of nanomaterials.^[^ ^72^ ^]^ Among them, the most common cation exchange technology uses Cu_x_S as the exchange template to obtain HENMs consistent with the original nanostructures through the exchange of copper ions and target metal ions at high temperatures.^[^ ^7^, ^23^ ^]^ A novel PEC immunoassay was constructed by using the hollow nanocube (ZnCdFeMnCu)_x_S photoactive matrix through cation exchange technology, which can realize the sensitive detection of prostate specific antigen (PSA)^[^ ^23b^ ^]^ (Figure 2G,K). In addition, topochemical transformation of Cu nanotubes was achieved by template‐triggered reduction alloying technique. Precisely regulated synthesis of PtPdRhCuM (M = Mn, Co or Ni) HEA nanotubes (NTs) with significant lattice distortion and abundant defect sites.^[^ ^39^ ^]^ Using this HENMs as an ultra‐sensitive sensor layer to determine the concentration of phenacyl sulfone, it shows that it has an ultra‐low detection line. It is worth mentioning that selective dealloying by acid‐base etching provides an effective means to create complex structural morphology, increase specific surface area and expose more active sites of HENMs.^[^ ^73^ ^]^ Therefore, topochemical transformation method has great advantages in constructing HENMs with specified nanostructure and element composition, especially in the process of model construction based on theoretical calculation after obtaining new materials, and in the study of material properties.

At the same time, combined with machine learning algorithm, we can further explore and design HENMs^[^ ^74^ ^]^ with specific properties and functions, greatly broaden its application boundaries, from energy, catalysis to biomedical and other fields, have shown broad application prospects (**Table** **2**).

**Table 2:** Summarized HENMs in different synthesis methods.

Method		Material	Morphology	Application	Refs.
	High thermal pyrolysis	MnFeCoNiCu	Nanosphere	Biosensor and antibacterial	[5a]
Bottom‐up method	Template assisted	PtPdMnCoFe	Nanochain‐like internetwork	Biosensor	[37]
	Sol‐gel assisted	PtPdRuRhIr	US‐nanoparticle	Antitumor	[25]
	Liquid‐phase exfoliation	TiVNbMoC_3_	Nanosheet	Antibacterial and tissue repair	[43]
Top‐down method	Ball milling	TiVCrMoC_3_	Nanosheet	Antibacterial	[21c]
	Laser ablation	HfNbTaTiZr	Nanosphere	Antitumor and antibacterial	[70]
Topochemical transformation	Cation exchange	(ZnCdFeMnCu)_x_S	Nanocube	Biosensor	[23b]
	Reduction alloying	PtPdRhCuM (M = Mn, Co or Ni)	Nanotube	Biosensor	[39]

## 4 The Development of HEMs in Biological Field

**Figure 3:**
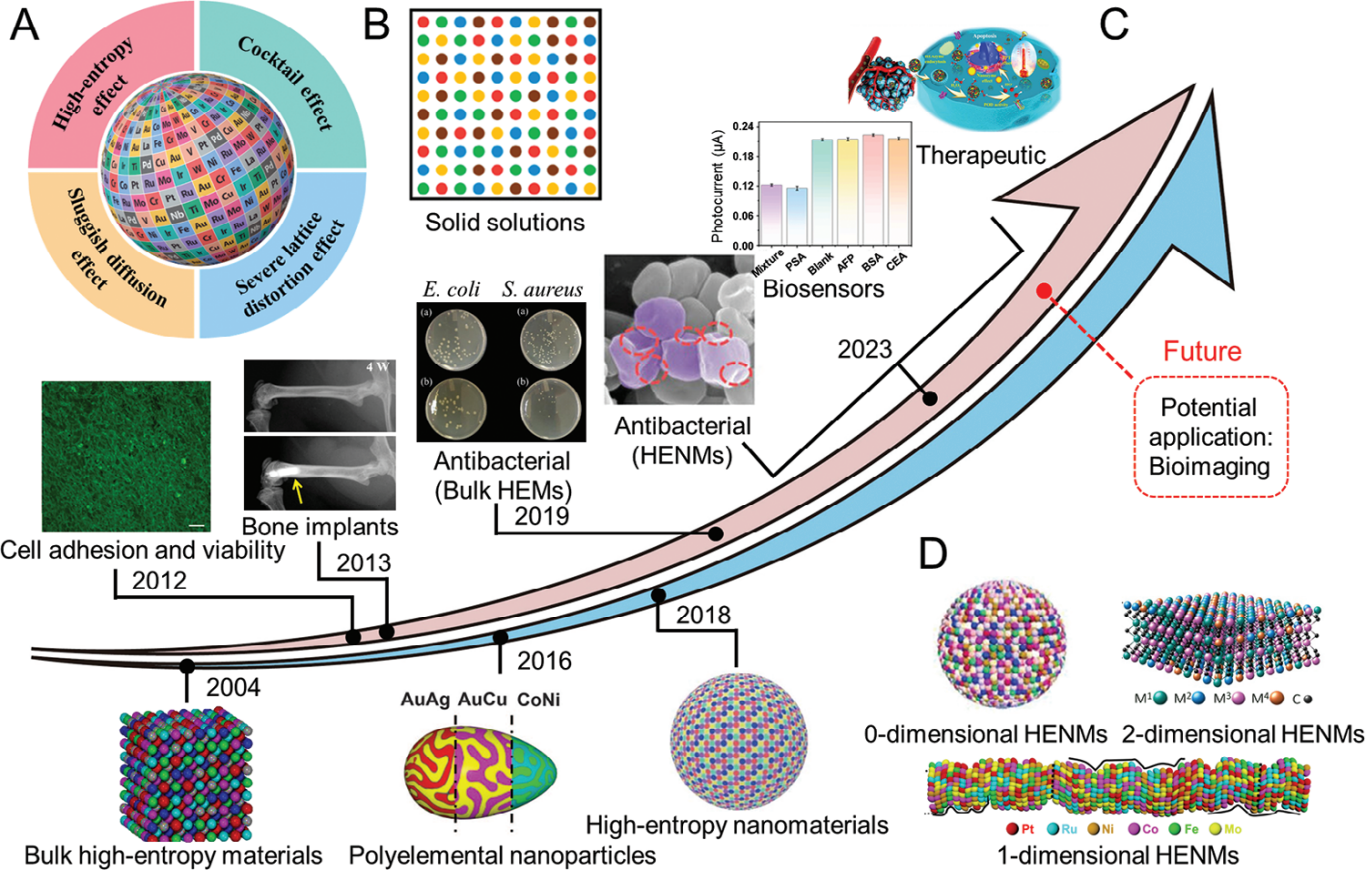
A) Four characteristics of high‐entropy materials.^[^ ^45b^ ^]^ Copyright 2024, Springer nature. B) A disordered structure diagram of an ideal random solid solutions.^[^ ^16^ ^]^ Copyright 2021, AAAS. C) The timeline of development of High entropy materials in biology (pink) and Bulk high entropy materials to high entropy nanomaterials (blue). Cell Activity and Adhesion on Surfaces of high entropy Materials.^[^ ^8c^ ^]^ Copyright 2012, Elsevier; Radiographs of mice distal femora with and without high‐entropy bone implants.^[^ ^29^ ^]^ Copyright 2013, Elsevier; Images of *S. aureus* and *E. coli* of incubation: a) control and b) AlCoCrCuFeMoNi.^[^ ^77^ ^]^ Copyright 2019, Cambridge University Press; Typical SEM image of MRSA bacterial after HENMs + H_2_O_2_ treatment.^[^ ^5a^ ^]^ Copyright 2023, John Wiley and Sons; PSA selectivity based on (ZnCdFeMnCu)xS immune sensor.^[^ ^23b^ ^]^ Copyright 2023, Elsevier; Ultra‐Small high entropy nanoparticles for tumor therapy.^[^ ^25^ ^]^ Copyright 2023, John Wiley and Sons; The emergence of bulk high‐entropy materials^[^ ^3^ ^]^ (Copyright 2004, John Wiley and Sons) to polyelemental nanoparticles (phase separation)^[^ ^11^ ^]^ (Copyright 2016, AAAS) to the high‐entropy nanoparticles^[^ ^12^ ^]^ (Copyright 2018, AAAS). Bioimaging is meant to be present at some time in the future. D) Schematic diagram of low‐dimensional and HENMs (0D,^[^ ^65^ ^]^ Copyright 2022, American Chemical Society; 1D,^[^ ^20a^ ^]^ Copyright 2021, Springer nature; 2D,^[^ ^21a^ ^]^ Copyright 2022, American Chemical Society).

Early research on HEMs focused on improving the mechanical properties of metal alloys as engineering materials,^[^ ^75^ ^]^ and until recently, the application of HEMs has gradually moved into the biomedical field due to their unique properties (**Figure** **3**C). HEMs have been investigated as a possible biomaterial based on their high mechanical properties and biocorrosion resistance, and related cytocompatibility research was conducted in 2012.^[^ ^8c^ ^]^ And by 2013, HE bulk metallic glass was actually being used as a bone implant for exploratory experiments.^[^ ^29^ ^]^ This shows that people are still cautious when considering the application of new materials in the biomedical field. Although a multi‐element mixture was used for antibacterial use in 2012,^[^ ^76^ ^]^ the true sense HEMs for antibacterial use was not investigated until 2019.^[^ ^77^ ^]^ Subsequently, reports gradually appeared on antimicrobial applications utilizing the special electronic structure of HEMs and the characteristics of designed ion dissolution. Despite years of development of HEMs, their biological applications are still facing bottlenecks, and the progress of bulk materials is slow, and it is difficult to break through their excellent functional potential. However, the emergence of HENMs was a turning point, and since 2018, its application has mushroomed into multiple frontiers of bioscience, rapidly breaking the original limitations.^[^ ^12^ ^]^ Nanozymes for diagnosis and treatment,^[^ ^5a^ ^]^ catalysts for biosensors,^[^ ^23b^ ^]^ and light absorption‐based methods for tumor treatment^[^ ^25^ ^]^ are expected to be rapidly developed by 2023. Obviously, HEMs can be applied in a very short period of time, due to improvements in nanotechnology, making it possible to prepare HENMs with specific structures and properties. However, the demand for high‐performance functional nanomaterials in the biomedical field has driven researchers to carry out in‐depth explorations of HENMs.

In the process of exploring the application of HEMs in the biomedical field, high‐entropy ceramics or coating other materials with it can show good biocompatibility in order to further improve the application safety of HEMs and expand the application scenarios of HEMs.^[^ ^1^, ^8^ ^]^ This makes bone implant materials no longer limited to bulk HEMs, but also reduces the difficulty of preparing high‐entropy bone implant materials. Whether it is HEMs or other types of functional materials, the biosafety in the biological application process is the first consideration. Therefore, the selection of natural biomolecules or artificial organic molecules with good biocompatibility to modify nanomaterials can further improve the biosafety of HEMs.^[^ ^30^, ^78^ ^]^ In addition to the intrinsic properties of the material, the structural characteristics of the material can also play a crucial role in the application process. For example, 2D high‐entropy MOF^[^ ^79^ ^]^ and mesoporous HENMs^[^ ^19^, ^60^ ^]^ may be directly delivered to lesions via loading drugs, to realize the integration of drug loading and targeted therapy. Adjusting the elemental composition of HEMs may also increase biocompatibility. In addition to Ti and Si, other elements have proven to be biocompatible, including Zn, Fe, Mo, Ni, Co, and Cr.^[^ ^42^, ^74^ ^]^ First, these elements do not affect the functioning of normal cells, and second, they can be used as components of certain enzymes or functional small molecules in living organisms. Many unique functional materials can be obtained by the nanization of bulk high‐entropy materials and the construction of various low‐dimensional nanometer structures (Figure 3D).^[^ ^44^, ^68^, ^80^ ^]^ Specifically, the effect of disordered polyvalent elements on lattice distortions and local electronic structures during the dedimensionalization process of high‐entropy bulk materials will affect the properties of HENMs.^[^ ^80d^ ^]^ Therefore, the nanomaterials obtained from the low‐dimensionalization of bulk high‐entropy materials must undergo strict performance evaluation before they can be applied in biology to prevent unnecessary negative effects caused by changes in physical and chemical properties. Notably, the high‐entropy interface of HENMs prompts the cell membrane to interact with the HENMs and improve the uptake capacity of the cells.^[^ ^57^ ^]^ As a result, HENMs can better adapt to the microenvironment in organisms and improve biocompatibility and functional performance, which has not been demonstrated by other materials. This approach may further provide opportunities for the expansion of HENMs applications in the biological field.

## 5 Application of HEMs in Biomedical Field

Although the current exploration of functional HEMs in the biomedical field remains in its infancy, HEMs, especially HENMs, have demonstrated fascinating properties due to their unique ability to accurately select functional elements and regulate their microstructure. HEMs have started to emerge in areas such as biosensors, antibacterial materials, therapeutics, bioimaging, and tissue engineering, showing great application potential and broad prospects.

### 5.1 Biosensors

Due to their high mixing entropy characteristics, HEMs offer efficient surface electron transfer and chemical reactivity, significantly accelerating the adsorption and reaction process of target analytes. This advantage has greatly improved the sensitivity of these biosensors, allowing them to achieve the accurate detection of low‐concentration target analytes.^[^ ^81^ ^]^ Therefore, HENMs offer great potential as a biosensing material for the real‐time monitoring of subtle changes in biomolecules or biological processes. In addition, the modification of different recognition elements (antibodies, DNA, and enzymes) on the surfaces of HENMs may significantly improve the sensitivity and selectivity of the biosensors for the detection of target molecules. This can facilitate the construction of biosensors based on changes in electrochemical or optical signals to achieve the precise quantitative analysis of target analytes such as proteins, DNA, ions, and pH values.

**Figure 4:**
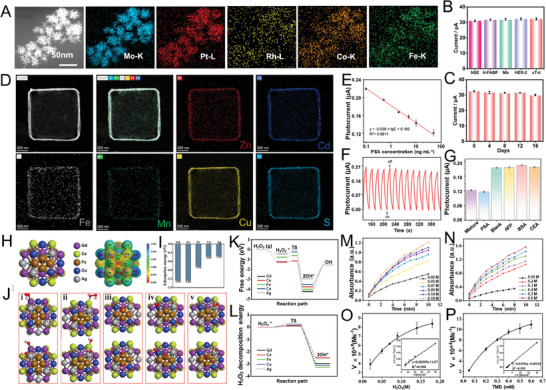
A) The HAADF‐STEM image and corresponding elemental mappings of HEANDs. B) The peak currents of the sensor without and with 50 ng mL^−1^ NSE, H‐FABP, Mb and HER‐2 at 1.0 ng mL^−1^ cTnI. C) The stability test at 1.0 ng mL^−1^ cTnI.^[^ ^5c^ ^]^ Copyright 2024, Elsevier. D) Elemental mapping of (ZnCdFeMnCu)_x_S nanocubes. E) Corresponding calibration curve based on (ZnCdFeMnCu)_x_S immune sensor. F) Stability of (ZnCdFeMnCu)_x_S immunoassay sensor‐based detection (PSA 0.5 ng mL^−1^). G) Selectivity based on (ZnCdFeMnCu)_x_S immune sensor (PSA 50 ng mL^−1^).^[^ ^23b^ ^]^ Copyright 2023, Elsevier. H) Optimized structure and calculated ESP of FeCuAgCeGd–HEAzyme. I) Calculated adsorption energy of H_2_O_2_ on at Fe, Cu, Ag, Ce, Gd sites, respectively. J) Optimized structures of H_2_O_2_ and ·OH adsorbed at i) Gd, ii) Ce, iii) Fe, iv) Cu, and v) Ag sites. K) Calculated Gibbs free energy change diagram from H_2_O_2_ to ·OH on FeCuAgCeGd–HEAzyme. L) Calculated H_2_O_2_ decomposition energy barrier on FeCuAgCeGd–HEAzyme. M) and O) Kinetics for POD–like activities of FeCuAgCeGd–HEzyme with different concentrations of H_2_O_2_; N) and P) Kinetics for POD–like activities of FeCuAgCeGd–HEzyme with different concentrations of TMB.^[^ ^23a^ ^]^ Copyright 2024, Elsevier.

Biosensing materials have mainly used the cocktail effect of HENMs to detect disease markers or drug metabolites through electrochemical immunoassays. For example, PtRhMoCoFe utilizes high‐entropy alloyed nano‐dendrites (**Figure** **4**A) as the electrochemical label‐free aptasensor. PtRhMoCoFe can be constructed using synergy between multiple metals and structural properties to realize the ultrasensitive determination of biomarker cTnI, enabling the early diagnosis and treatment of tumors (Figure 4B,C).^[^ ^5c^ ^]^ A novel electrochemical amperometric immunosensor has been developed for ultrasensitive detection of tumor marker neuron‐specific enolase (NSE), utilizing a self‐supporting PtPdMnCoFe high‐entropy alloy with a nanochain network as an efficient signal amplifier.^[^ ^37^ ^]^ In a refined analytical setting, the sensor exhibited an impressive low detection limit (LOD) of 0.0036 pg mL^−1^, with a signal‐to‐noise ratio (S/N) of 3, across the linear range spanning from 0.1 pg mL^−1^ to 200 ng mL^−1^. achieving the quantitative detection of human serum samples. This approach demonstrated the great potential application value of HENMs in biosensors and immunoassays.

In addition to their excellent cocktail effect, their highly customizable electrochemical properties are unique to high‐entropy materials, providing high stability and sensitivity in photoelectrochemical (PEC) immunoassays. Nano‐sulfide (ZnCdCuAgCo)_x_S semiconductors with a controlled morphology can be obtained through a cation exchange strategy, providing the sensitive and accurate detection of prostate‐specific antigen (PSA).^[^ ^7b^ ^]^ The results of clinical serum samples obtained by this PEC immunoassay were found to be very consistent with those obtained by enzyme‐linked immunosorbent assays. In another study, a novel hollow nano‐cube (ZnCdFeMnCu)_x_S (Figure 4D) photoactive matrix with a high‐entropy effect was constructed, also using cation exchange for the PEC immunoassay of PSA (Figure 4E–G).^[^ ^23b^ ^]^ This confirmed the excellent synergistic relationship between multiple elements in HENMs and also demonstrated that cation exchange technology could serve as an effective strategy to achieve continuously tunable HENMs with specified structures and compositions.

Point‐of‐care testing (POCT) has played a vital role in clinical applications, especially in the face of the COVID‐19 epidemic. Nanozyme‐based colorimetric platforms have been identified as a potential strategy for POCT, among which colorimetric sensors with polymetallic (FeCuAgCeGd)‐HEAzyme as ultra‐fine coordination nanoparticles (≈5 nm) as the core material have been reported for the detection of dopamine (DA).^[^ ^23a^ ^]^ The POD‐like activity of FeCuAgCeGd‐HEAzyme was confirmed through density functional theory (DFT) computations, exhibiting its capacity to facilitate the conversion of H_2_O_2_ into hydroxyl radicals (·OH) (Figure 4H–L). This verified that HENMs can exhibit high stability and good selectivity (Figure 3M–P), and enable fast, visual, and field detection in clinical applications.

By leveraging the mutual modulation among diverse atoms in high‐entropy nanomaterials, researchers have capitalized on high‐entropy oxides (HEO) composed of five metallic elements (Ni, Co, Cr, Cu, and Fe) to expedite the reduction of luminol‐dissolved O_2_ (DO) into reactive oxygen species (ROS), thereby enhancing the electrochemiluminescence (ECL) performance of the luminol‐DO system.^[^ ^6b^ ^]^ The unique crystal structure of HEO gives rise to abundant oxygen vacancies, facilitating the efficient conversion of DO into ROS and subsequently boosting the performance of the corresponding ECL sensor by ≈240‐fold. The HEO‐incorporated luminol‐DO ECL sensing system has been effectively utilized in the efficient biosensing of dopamine and alkaline phosphatase. This demonstrated that the unique electron transfer and catalytic properties of the surface of HENMs offer a significant advantage in sensor design.

In addition to the naked HENMs directly used in the preparation of biosensors, the surface‐anchored response recognition elements can be used to achieve the precise quantitative detection of a substance. PtPdRhCuM (M = Mn, Co, or Ni) HE nanotubes have been used as ultrasensitive sensing layers for the determination of phenacyl sulfone due to their composition diversity, high mixing entropy, inherent electronic conductivity, and surface structure complexity.^[^ ^39^ ^]^ The biosensor utilizing acetylcholinesterase‐Nafion/PtPdRhCuMn HE nanotubes exhibited a broad detection range, spanning from 0.45 picomolar (pM) to 45 nanomolar (nM), coupled with an impressively low detection limit of 86.5 femtomolar (fM). Extremely high recovery rates were also obtained when used for the detection of pesticides in actual food samples (apples and cucumbers).

Therefore, through fine elemental composition fine‐tuning and surface specific modification of HENMs, a large number of active sites are cleverly introduced on the surface of HENMs. These sites can interact closely and efficiently with biomolecules under test through various mechanisms such as chemical bonding, hydrogen bonding network and electrostatic interaction. This interaction process directly leads to significant changes in the photocurrent signal, providing a sensitive response for the detection of biomolecules. Further, with the help of advanced signal amplification technology, these subtle photocurrent changes can be accurately captured and amplified to achieve highly selective identification and efficient detection of biomolecules. This process not only improves the accuracy of the detection, but also significantly enhances the sensitivity and response speed of the sensor. In summary, the carefully designed and synthesized HENMs has shown unparalleled advantages in the field of biosensors: high sensitivity ensures the accurate capture of trace biomolecules, highly selective elimination of interference factors, and fast response to meet the needs of real‐time detection. These characteristics indicate that the application of HENMs in the field of biosensors will have a very broad development prospect, and contribute an important force to promote biomedical research and technological progress.

### 5.2 Antibacterial Activity

The potential application value of HEMs in the antibacterial field has been explored through different strategies, including the tight adsorption of bacteria with high surface activity, designed ion dissolution, and the induction of ROS production.

**Figure 5:**
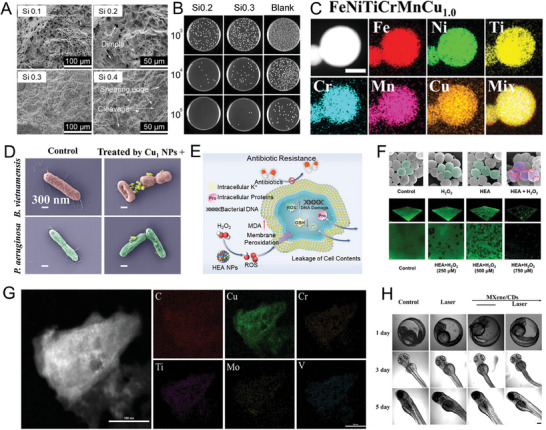
A) Fractured morphologies of Si0.1–Si0.4. B) Bacterial colonies after cultivation on Si0.2, Si0.3 and blank specimens.^[^ ^84^ ^]^ Copyright 2022, Elsevier. C) TEM‐EDS maps of the composing elements of FeNiTiCrMnCu_1.0_ HENMs. D) SEM mappings of *P. aeruginosa* incubated with Cu_1.0_ HENMs at a concentration of 600 µg mL^−1^ under light irradiation.^[^ ^33^ ^]^ Copyright 2023, John Wiley and Sons. F) Principle for ROS‐mediated inactivation of drug‐resistant bacteria based on the HENMs. G) Typical SEM images of MRSA after different treatments. H) 3D CLSM images of biofilms formed by MRSA with different treatments.^[^ ^5a^ ^]^ Copyright 2023, John Wiley and Sons. I) EDS elemental maps of HEMXene/CDs. J) Analysis of zebrafish development in different treatment groups.^[^ ^44^ ^]^ Copyright 2024, Elsevier.

Early studies explored the inhibition of microbial growth on the surface of high‐entropy alloying bulk materials or HEMs coatings. Studies demonstrated that high‐entropy materials containing Cu and Ag exhibited significant antibacterial activity, with Zr and Al insensitive to antibacterial activity.^[^ ^82^ ^]^ Notably, HEMs containing copper (Cu‐HEAs) exhibited highly effective anti‐viral properties. For example, Cu‐HEAs surfaces were able to inactivate more than 99.99% of influenza viruses H1N1 and enterovirus 71 and were no longer infectious after 24 h of treatment.^[^ ^83^ ^]^ In the antibacterial process of a series of Cu‐containing CrFeNiCuSi_x_ (x = 0.2, 0.3), HEAs without expensive Co showed that an increase in Si content led to the strengthening of HEAs, and the corrosion resistance was closely related to the distribution of Cu (**Figure** **5**A,B).^[^ ^84^ ^]^ In addition, Fe‐rich and Co‐free Fe_1.3_CrNiCu_0.7_Si_0.2_ HEAs showed a bacteriostasis rate of up to 98% against *Escherichia coli*.^[^ ^85^ ^]^ While ensuring an antibacterial effect, the cost of high‐entropy antibacterial materials was reduced by using low‐cost metal elements to design and replace high‐cost elements in the high‐entropy material, which reflected the advantages of the flexible adjustment of multiple elements in the HEMs.

Although HEMs have the characteristics of mechanical stability, the designed ion dissolution characteristics of high‐entropy antibacterial materials may be endowed by adjusting the proportion of elements, serving as the basis for the diversification of use scenarios of the HEMs. For example, through the design and preparation of Co_0.4_FeCr_0.9_Cu_0.3_, antibacterial HEAs could achieve *Escherichia coli* and *Staphylococcus aureus* bacteriostasis rates of 99.97% and 99.96% after 24 h, respectively, which was better than the bacteriostasis rate of classic copper containing stainless steel. ROS analysis results showed that the release of Cu ions and direct contact with the Cu‐rich phase had a synergistic effect in enhancing antibacterial properties. This provided new insights into the design of structure‐function‐integrated antibacterial alloys.^[^ ^24^ ^]^ In addition, a copper containing Al_0.4_CoCrCuFeNi HEA was produced with a broad antibacterial spectrum and strong mechanical properties. The high concentration of copper ions released by this HEA could hinder the growth of bio‐corrosive marine bacteria and the formation of biofilms.^[^ ^86^ ^]^ Compared with traditional antibacterial alloys, the rational design of this structure provided justification for the further development of unique HEA materials with high antibacterial efficiency and mechanical properties. Antibacterial HEMs may be able to achieve a high antibacterial effect through the design dissolution of antibacterial metal ions while also ensuring high mechanical structural strength through the design of a unique element ratio.

When HEMs enter the nano‐scale era, this will not only provide significant cost savings for antibacterial materials but also further broaden the application of antibacterial applications. As a result, the application of HENMs has gradually become an economically feasible and effective anti‐infection treatment method. In an era of high drug resistance, innovation, new antimicrobial agents, and antimicrobial strategies are needed to control infection. Nano‐scale high‐entropy bacteriostatic materials can better exert their physicochemical properties and structural particularities. The development of multi‐modal antimicrobial strategies may be used to effectively sterilize and prevent drug resistance. For example, FeNiTiCrMnCu_x_ HENMs with excellent photothermal heating properties, assisted by high‐entropy effects, have demonstrated high efficiency of combined antibacterial elements and non‐contact heating capabilities toward antimicrobial and anti‐biofilm activity (Figure 5C,D).^[^ ^33^ ^]^ Under the synergistic action of Cu ions released by HENMs and thermal damage, more ROS were produced, leading to cell membrane rupture and biofilm destruction. Experiments demonstrated that the antimicrobial membrane efficiency (400 µg mL^−1^) of optimized FeNiTiCrMnCu_1.0_ HENMs in marine nutrient media could be increased from 81% to 97.4% after 30 min of solar irradiation. This serves as a typical case of achieving multimodal resistance against drug‐resistant bacteria.

Mixing a variety of transition metals can enable HENMs with high enzymatic catalytic activity. For example, a class of MnFeCoNiCu transition metal high‐entropy nanozymes with strong substrate affinity and high catalytic efficiency can produce ROS such as ·OH, ·O_2_^−^, and ^1^O_2_ (Figure 5E,F) when mixed with H_2_O_2_.^[^ ^5a^ ^]^ High‐entropy nanozymes possess distinct surface atomic configurations and unique D‐orbital coupling features among their various metal components. Detailed DFT calculations revealed that the enhanced D‐orbital coupling among these metals led to an increase in electron density proximate to the Fermi energy level (EF) and a shift in the overall position of the D‐band center relative to EF. This enhancement not only improved the efficiency of localized electron transfer but also boosted the adsorption of oxygen intermediates during the catalytic process. These HENMs demonstrated remarkable substrate affinity and catalytic efficiency, rivaling that of natural horseradish peroxidase (HRP). Therefore, HEzyme, with its outstanding POD‐like activity, holds promising applications in the fields of biosensing and antimicrobial technology.

In addition to 0D HENMs, 2D high‐entropy MXenes (HEMXenes), which expose the active sites to a high‐entropy atomic layer, offer significant advantages in terms of antimicrobial infection. By utilizing single‐layer HEMXenes, which are fabricated from transition metals exhibiting high entropy and low Gibbs free energy, it is feasible to achieve high‐performance hyperthermia and inherent biocatalysis, thereby effectively treating drug‐resistant bacterial infectious diseases.^[^ ^21c^ ^]^ With increased entropy, HEMXenes exhibit extremely strong simulated oxidase activity and photothermal conversion efficiency of 65.8% in the second near‐infrared (NIR‐II) biological window. Monolayer HEMXenes as therapy nano‐agents can effectively treat BK and subcutaneous abscess infections caused by MRSA, with good efficacy in promoting the healing of infected tissues. In addition, HEMXene‐TVNMC as a sound sensitizer can be used to generate ROS against MRSA.^[^ ^43^ ^]^ Interestingly, the energy storage and conversion of TVNMC MXenes with different compositions allowed for ROS production under US irradiation and even enhanced ROS under photothermal therapy. In addition, TVNMC MXenes promoted wound healing while eliminating MRSA abscesses under both acoustodynamic and photothermal therapies. An antibacterial strategy of HENMs to promote the production of h^+^/e^−^ has also been investigated through the injection of external energy in high‐entropy semiconductor materials and inducing the production of ROS to achieve an antibacterial effect.

The design of heterojunction structure can inhibit the rapid recombination of h^+^/e^−^ produced in narrow band gap semiconductors to further improve the antibacterial effect. HEMXene/CD heterojunction nanomaterials can be used for oxidative stress amplification under NIR laser irradiation (Figure 5G).^[^ ^44^ ^]^ Well‐spalling MXene nanomaterials were found to exhibit good bactericidal photothermal effects. The addition of Cd can provide photogenerated electrons for HEMXene nanosheets to generate ROS while reducing the recombination of h^+^/e^−^ pairs and accelerating the generation of photogenerated electrons. MXene/CDs achieved 99.99% killing efficiency against resistant *Escherichia coli* and *Staphylococcus aureus* through synergistic photothermal and photodynamic effects. The incubation and growth process of zebrafish demonstrated the biosafety of the heterojunction materials (Figure 5H). In addition to the design of the intrinsic properties of HENMs, the rational design of the microstructure may expand its antibacterial properties.

It can be seen that the designed ion dissolution mechanism, semiconductor photogenerated e^−^/h^+^ pairs, thermal damage and ROS generation can destroy the bacterial cell barrier (cell wall, cell membrane) or inhibit its normal growth to varying degrees, and then make the bacteria lose external support and die due to osmotic pressure change. Because of the existence of bacterial cell wall, HENMs are difficult to enter the bacteria and cause great damage to its contents.

### 5.3 Antitumor Therapeutics

Although HENMs exhibit significant potential in the therapeutic field due to their unique excellent properties, current application cases remain rare. However, the few available cases reflect the unique characteristics of HENMs in this field. For example, the PTT and PDT of tumors can be realized through the photo‐effect of HENMs, the Fenton‐like effect can be realized using materials containing variant metal elements, and semiconductor properties can kill tumor cells through hole and electron separation. In addition, the combination of drug loading and multiple treatment methods serves as a common treatment strategy.

**Figure 6:**
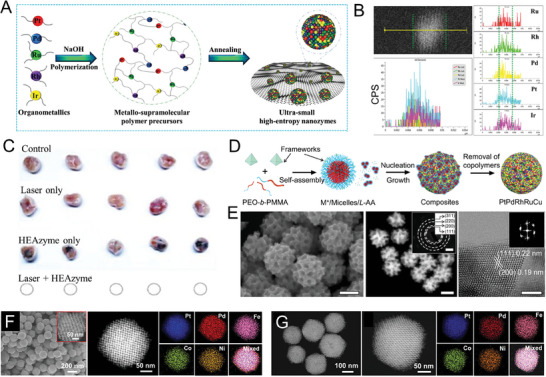
A) Fabrication of US‐HENMs by metal‐ligand cross‐linking strategy: the organometallics turn to polymer precursors through the aldol condensation reaction, and the polymer precursors finally turn to the US‐HENMs by annealing treatment. B) Linear scan characterization images of the US‐HENMs. C) Images of tumor obtained from different treated groups.^[^ ^25^ ^]^ Copyright 2023, John Wiley and Sons. D) Schematic illustration of the synthesis of PtPdRhRuCu HENMs. E) SEM, HAADF–STEM (scale bar: 100 nm). Inset: The corresponding SAED pattern (scale bar: 5 nm^−1^), and HRTEM (scale bar: 2 nm) (FFT patterns was shown inset).^[^ ^19^ ^]^ Copyright 2023, Springer Nature. F) Structural characterization of MHEI‐PtPdFeCoNi and MHEA‐PtPdFeCoNi including low‐magnification SEM and HAADF‐STEM. G) high‐magnification HAADF‐STEM and corresponding EDX mapping images.^[^ ^60b^ ^]^ Copyright 2023, John Wiley and Sons.

As a photothermal agent, HENMs provide a precise and localized approach to targeted cancer cells and bacterial ablation through photothermal therapy. This approach uses visible or NIR light to generate local heat for therapeutic purposes.^[^ ^53a^ ^]^ Ultra‐small HENMs, such as PtPdRuRhIr US‐HENMs, have been used as a bi‐functional nanoplatform for the efficient treatment of tumors (**Figure** **6A–C**).^[^ ^25^ ^]^ US‐HENMs, such as PtPdRuRhIr, not only exhibit excellent POD‐like activity to catalyze endogenous H_2_O_2_ to produce highly toxic ·OH radicals but also exhibit a high photothermal conversion ability to convert 808 nm NIR light into heat energy. 2D Cu_2_Zn_1_Al_0.5_Ce_5_Zr_0.5_O_x_ HENMs have potential application value in photothermodynamic therapy.^[^ ^21b^ ^]^ The HENMs can effectively kill cancer cells through local irradiation while reducing damage to normal cells. To develop an efficient and stable photothermal therapeutic agent, researchers have successfully prepared HfNbTaTiZr refractory high‐entropy alloy colloidal nanoparticles through the pulsed laser ablation of the liquid medium under different conditions.^[^ ^70^ ^]^ The NPs produced in ethanol were irradiated at 640 nm for 20 min, and the temperature increased by 12 °C, which fully demonstrated the significant potential of HfNbTaTiZr HENMs for tumor photothermal therapy. Nanomaterials with a certain morphology and structure often have different therapeutic effects compared with simple spherical nanoparticles in the application process. For example, 1D FeCoNiCuZn high‐entropy nanowires containing magnetic and non‐magnetic elements have shown significant PTT efficacy, with a potential application value of magnetothermal therapy (MHT).^[^ ^5d^ ^]^ These magnetic elements make them a dual material, allowing them to be used as an operative agent for both PTT and MHT jointly or separately. Some of the materials mentioned in the previous section for antibacterial applications can also be used in tumor therapy using a similar bacterial killing mechanism.

As a non‐invasive cancer treatment, MHT has the characteristics of strong penetration, minimal damage to surrounding normal tissue, and no multi‐drug resistance, offering great application prospects. To obtain magnetic nanoparticles with high magnetothermal efficiency, researchers have developed a class of high‐entropy magnetic nanoparticles containing FeCoNiCu (HEMNPs) with special magnetothermal properties synthesized by the thermal decomposition method.^[^ ^36^ ^]^ The fabricated HEMNPs exhibited impressive magnetic properties, including a saturation magnetization value of 42.4 emu g^−1^ and a coercive force of 129.5 Oe. When subjected to an alternating magnetic field with an intensity of 46 Oe and a frequency of 266 kHz, these nanoparticles achieved a remarkable maximum specific power loss of 202 W g^−1^ at a concentration of 2 mg mL^−1^. This outstanding performance suggests significant potential for their application in the realm of magnetic therapy (MT).

The combination of functional HENMs and high‐entropy nanocarriers with a good carrying capacity for drugs can allow the drug to reach the lesion site more effectively while realizing this therapy. The combination of multiple therapeutic methods may also improve the therapeutic effect while reducing damage to normal tissue. For example, high‐entropy nanospheres with an exposed mesoporous structure PtPdRhRuCu (Figure 6D and E),^[^ ^19^ ^]^ polyhedral hollow high‐entropy metal oxide ZnFeNiCuCoRuO,^[^ ^60a^ ^]^ porous nanowires (PtPdRhIrNi and NiPtPdRhIrAl),^[^ ^73^ ^]^ ordered mesoporous spheres (Figure 6F and G),^[^ ^60b^ ^]^ high‐entropy mesoporous spheres,^[^ ^6^, ^59^ ^]^ and other porous materials can achieve drug spot release and chemocatalysis after loading related drugs. Compared with traditional drug carriers, HENMs can form stable composite structures with drug molecules due to their biocompatibility and adhesion and can also control their motion and drug release at specific locations through a light field, magnetic field, or other means.

In summary, the application of HENMs in the field of tumor therapy is showing its excellent potential in a diversified mechanism. First of all, they can efficiently convert light, sound, magnetic and other external energy into heat energy, and accurately act on tumor tissues to achieve local high‐temperature destruction, which not only improves the selectivity of treatment, but also reduces the potential damage of traditional hyperthermia to surrounding healthy tissues. Second, HENMs show unique advantages in regulating tumor microenvironment. By generating ROS (such as ·OH, ^1^O_2_, O_2_·^−^) and other reactive oxygen species, they can effectively break the REDOX balance in tumor cells and induce programmed cell death such as iron death and pyrodeath. In addition, the regulation of ROS also profoundly affects the metabolism and signal transduction of tumor cells, further inhibiting tumor growth and metastasis, and providing strong support for tumor treatment. Moreover, HENMs are ideal carriers for anti‐tumor drug delivery due to their unique structure and surface properties. They can achieve targeted delivery and controlled release of drugs, significantly improve the bioavailability and therapeutic effect of drugs, and reduce the toxic side effects of drugs, bringing a safer and more effective treatment experience for patients. Therefore, the application of HENMs in tumor therapy has a variety of mechanisms and advantages, and provides a new idea and method for tumor therapy. In the future, with the deepening of research and the development of technology, the application prospect of HENMs in the field of tumor therapy will be broader.

### 5.4 Bioimaging

The pursuit of high sensitivity, spatio‐temporal resolution, and non‐invasive multi‐modal bioimaging technology serves as an important means of medical research and clinical diagnosis and treatment. However, most nanomaterials only have a single bioimaging capability, making it challenging to collect accurate disease‐related data.^[^ ^87^ ^]^ The ability of HENMs to act as a contrast agent for bioimaging by adjusting the internal composition and surface functionalization makes multimodal imaging easy to achieve, and combining different imaging techniques, such as magnetic resonance imaging, X‐ray imaging, and optical imaging, can be used to obtain more comprehensive and precise biological information. In addition, the basic principle of bioimaging technology and diagnosis relies on the various physical and chemical properties of contrast media materials with special properties to develop areas of interest.^[^ ^88^ ^]^ Therefore, the design and preparation of the developer based on the basic principle of imaging is the most efficient.

**Figure 7:**
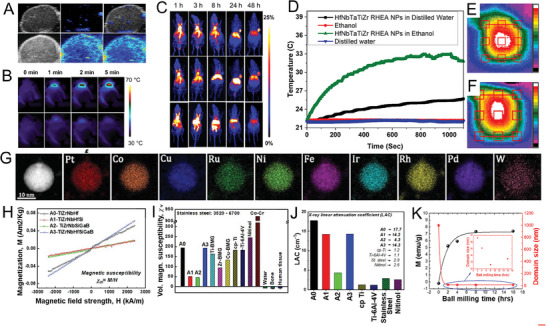
A) Au nanorings as PA contrast agent and photothermal therapeutics. US, PAI, and merged images of tumors (from left to right) before (upper row) and 24 h after (lower row) intravenous injection. B) Thermal images of U87MG tumor mice injected intravenously with 50 nm Au nanorings (upper) and PBS (lower) upon 5 min laser irradiation. C) Representative whole‐body coronal PET images of mice after intravenous injection of ^64^Cu‐labeled Au nanorings, Au nanospheres and Au nanoplates.^[^ ^92^ ^]^ Copyright 2017, American Chemical Society. D) Representative graph showing the measured variation of the temperature for the pure distilled water, pure ethanol, and produced HfNbTaTiZr HENMs in distilled water, ethanol as a function of time under 640 nm irradiation. E) The resulting steady‐state IR camera image of the produced HfNbTaTiZr HENMs in distilled water. F) The resulting steady‐state IR camera image of the produced HfNbTaTiZr HENMs in ethanol.^[^ ^70^ ^]^ Copyright 2023, Elsevier. G) Elemental maps of HENMs.^[^ ^65^ ^]^ Copyright 2022, American Chemical Society. H) The magnetization versus applied magnetic field recorded for A0, A1, A2, A3. I) Volume susceptibility values of studied MPEAs presented in comparison with other alloys and human tissues. J) Radiopacity in terms of X‐ray linear attenuation coefficient.^[^ ^50^ ^]^ Copyright 2021, Elsevier. K) Ball milling time dependence on saturation magnetization and the magnetic domain size of the samples.^[^ ^102^ ^]^ Copyright 2022, American Chemical Society.

Photoacoustic imaging (PAI) involves the use of the photoacoustic effect of the light absorber to scatter photons in tissue, providing significant imaging depth and higher spatial resolution than classical in vivo optical imaging.^[^ ^89^ ^]^ The photoacoustic effect occurs when a pulsed or modulated light source is used, and the temperature of the light absorber will cause its size to expand and contract, causing the sound waves to radiate outward.^[^ ^90^ ^]^ As a result, photoacoustic imaging and photothermal therapy are often integrated,^[^ ^91^ ^]^ even though the versatility and structural characteristics of special materials to achieve a variety of modes of imaging, including PA and PET imaging (**Figure** **7**A–C).^[^ ^92^ ^]^ Some HENMs exhibit the excellent absorption and scattering performance of light (Figure 7D–F),^[^ ^5^, ^70^ ^]^ which can be used as a contrast agent for biological imaging technology, to improve the resolution and contrast of the image. This can help scientists obtain a deeper understanding of the internal structure and function of the cell and monitor the cell activity state in real time.

Computed tomography (CT) and X‐ray imaging techniques both rely on the penetration properties of X‐rays into different tissues in the body and the resulting absorption and scattering effects to form images.^[^ ^93^ ^]^ CT contrast agents play a pivotal role in discriminating between tissues with comparable attenuation coefficients during these imaging procedures. Elements such as I, Au, Bi, and Xe^[^ ^94^ ^]^ exhibit high attenuation coefficients for X‐rays due to their high elemental numbers. In addition, 2D nanomaterials based on Pd have shown excellent CT imaging ability due to their high elemental number characteristics.^[^ ^27^, ^95^ ^]^ HENMs have attracted widespread attention in the field of materials science due to their unique atomic structure and physical properties. In the process of synthesizing high‐entropy materials, it may be possible to effectively enhance their absorption of X‐rays by introducing elements with high elemental ordinal numbers,^[^ ^37^, ^39^, ^59^ ^]^ thus giving them significant advantages as contrast agents for CT/X‐ray images. In addition, we can expect other Pt‐based HENMs to show more potential and opportunities in CT/X‐ray imaging applications (Figure 7G).^[^ ^60^, ^65^ ^]^

Magnetic resonance imaging (MRI), based on the spin of protons in the human body in the presence of an external magnetic field, can be stimulated by radio frequency pulses, providing high spatial resolution, temporal resolution, and excellent intrinsic soft tissue contrast.^[^ ^96^ ^]^ Currently, clinically approved MRI contrast agents mainly use gadolinium‐based (Gd III) complexes.^[^ ^97^ ^]^ In recent years, superparamagnetic iron oxide (SPIO) has been commonly used in this class of agents,^[^ ^98^ ^]^ with its main components consisting of magnetite (Fe_3_O_4_) or magnetite (Fe_2_O3). In addition, manganese‐based^[^ ^99^ ^]^ and superparamagnetic PtFe nanoparticles^[^ ^100^ ^]^ are used in MRI imaging. Target‐designed amorphous ultra‐low para‐magnetism HEMs can serve as candidates for achieving multimodal imaging (Figure 7H–J).^[^ ^50^ ^]^ Therefore, HENMs superparamagnetic materials may have potential applications in MRI imaging, including superparamagnetic HENMs (Figure 7K) obtained during the nanization of bulk materials.^[^ ^101^ ^]^ In addition, ultrasound (US), positron emission tomography (PET), and fluorescence imaging may be explored based on the comprehensive characteristics of HEMs.^[^ ^88b^ ^]^

To sum up, in order to design multifunctional nanocontrast agents with multimodal biomedical imaging capabilities, it is essential to establish nanoplatforms with their inherent composition, nanostructure and activity. For example, the synthesis of HENMs containing high elemental numbers, these materials with their unique X‐ray absorption properties, can produce clear gray scale images in CT scans, providing clinicians with detailed anatomical information; Photothermal conversion HENMs can rapidly convert light energy into heat energy after receiving a specific wavelength of light, resulting in thermal expansion of local tissues, which in turn generates ultrasonic signals, which makes them show great potential in the field of photoacoustic imaging (PAI), which can achieve non‐invasive, high‐resolution imaging of the internal structure of biological tissues. Superparamagnetic HENMs can produce a strong local magnetic field effect in the magnetic field environment, which can significantly shorten the relaxation time of hydrogen protons in the surrounding tissue, thus effectively enhancing the contrast of MRI images. This property makes superparamagnetic HENMs an ideal contrast agent for MRI imaging, which can help doctors more accurately identify diseased areas and improve diagnostic accuracy and reliability. Considering that the development time of HENMs is still short, we have reason to believe that with the deepening of research and the maturity of technology, it will show a more dazzling light in the field of biological imaging. Although the existing cases in biological imaging are not enough to fully reflect the powerful functions of HENMs, with the continuous advancement of scientific research, it will certainly play a more important role in this field in the future and make greater contributions to human health.

### 5.5 Tissue Engineering and Regenerative Medical

Compared with other aspects of research in the biomedical field, HEMs have been assessed in bone tissue engineering relatively early. Due to the increasing demand for artificial bone and bone implants to replace degraded bone in the human body, the demand for bone implants with excellent biocompatibility and mechanical properties in bone tissue engineering has continued to increase. With high hardness, good wear and corrosion resistance, and good biocompatibility, HEMs can be used in bone implant engineering and are considered the best alternative to biological scaffolds or biological materials.^[^ ^52b^ ^]^

Studies have shown that adjusting the content of constituent elements in HEMs can affect their microstructure, corrosion properties, and wear behavior.^[^ ^49^ ^]^ For example, the dendrite morphology and mechanical properties of TiNbTaZr and TiNbTaZrMo alloys can be controlled by adjusting the proportion of component elements. In addition, easily passivated elements such as Nb, Ta, and Mo can usually improve the chemical stability of the oxide film on the surface of the alloy.^[^ ^28a^ ^]^ Therefore, it is important to study the effect of component element content on the microstructure, corrosion, and wear behavior of TiZr refractory HEA systems. Compared with traditional bone implants, this excellent performance serves as a prerequisite for the wide application prospects of HEMs in the biological field.^[^ ^52^, ^103^ ^]^

**Figure 8:**
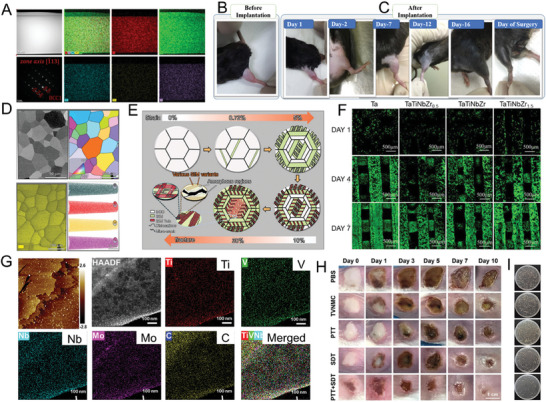
A) The SEM imaging and the TEM observations of the SEAD patterns and elemental mapping of (MoTa)_0.2_NbTiZr. B) Visual evidence of mice thigh before and C) after implantation (MoTa)_0.2_NbTiZr alloy.^[^ ^52a^ ^]^ Copyright 2021, Elsevier. D) Overview of microstructures of the ST Ta_10_ alloy via ECC imaging, EBSD IPF and EBSD phase maps, and 3D atom maps of Ti, Zr, Hf, and Ta in a typical tip containing the BCC matrix. E) Schematic diagram of microstructure evolution in the Ta_10_ Bio‐HEA during tensile deformation.^[^ ^38^ ^]^ Copyright 2024, John Wiley and Sons. F) Fluorescence images of MC3T3‐E1 cells cultured on porous TaTiNbZr scaffolds and the porous control Ta scaffolds for 1, 4 and 7 days.^[^ ^35^ ^]^ Copyright 2023, Elsevier. G) AFM micrograph and HAADF‐STEM of the monolayer TVNMC HEMXene indicates a uniform layered morphology and atomic distribution of the Ti, V, Nb, Mo, and C atoms. H) Images of MRSA‐infected abscesses from various groups at varying treatment times. I) Photographs of the MRSA colonies for the varying treatment groups.^[^ ^43^ ^]^ Copyright 2024, Elsevier.

In addition to the above properties, the yield strength of bone implants serves as a key parameter. A comparison of the use of medium‐ and high‐entropy (MoTa)_x_NbTiZr alloys in biomedical implants showed that the hardness and yield strength increased logarithmically with an increase in Mo and Ta content (Figure **8A–**C).^[^ ^52a^ ^]^ The plastic strain of the alloy with 0.4 mol Ta content was greater than 30% under compression, and the addition of Mo and Ta linearly increased the elastic modulus of the system. Among these, the (MoTa)_0.2_NbTiZr alloy with excellent biocompatibility, biocorrosion resistance, strength, and ductility can serve as a potential structural biomedical implant material. The toughness of the implant material can effectively alleviate the stress shielding effect and avoid stress concentrations at the interface between the implant and bone, possibly leading to implantation failure. Better fatigue resistance and impact resistance can also withstand various stress changes in the physiological environment and maintain the stability and integrity of the implant.

Although bone implant materials require a high modulus of elasticity, a high modulus of elasticity is not always ideal; therefore, balance between multiple properties of the material must be obtained.^[^ ^104^ ^]^ Compared with traditional alloys, the elastic modulus remains limited (45–210 GPa), with a large gap compared with human bone (≈30 GPa), and alloys with a low elastic modulus tend to have limited plasticity. Researchers have designed and prepared metastable BCC TiZrHfTa biological high‐entropy alloys (Figure 8D and E), composed of non‐toxic materials, with a low elastic modulus and high plasticity.^[^ ^38^ ^]^ The stress‐induced martensitic transformation of HEMs will occur during the tensile process, resulting in extremely high work hardening and good plasticity of the alloy.

The stability and adjustability of the HEMs can regulate the mechanical properties and biological activity of the biological scaffold, achieving good adhesion and biological activity of the scaffold and surrounding tissues and promoting tissue repair and regeneration. For example, a novel 3D‐printing technology was used to prepare a porous bio‐HEA scaffold (Figure 8F) using Ta, Ti, Nb, and Zr as raw materials.^[^ ^35^ ^]^ 3D scaffolds with porous structures were fabricated at ambient temperature and subsequently sintered to enhance their mechanical robustness. The interdiffusion of metal elements resulted in the creation of a porous biological HEA scaffold exhibiting a BCC structure. The compressive strength of these scaffolds ranged from 70.08 to 149.95 MPa, while their elastic modulus spanned from 0.18 to 0.64 GPa, allowing for extensive control over their mechanical properties. Notably, the compressive strength of the scaffold approximated that of human cortical bone, thus fulfilling the criteria for bone implants in terms of porous structural features and biomechanical performance. Furthermore, these scaffolds effectively promoted cell adhesion, proliferation, and tissue repair. The Ca_0.2_Mg_0.2_Zn_0.2_Sr_0.2_Yb_0.2_ high‐entropy bulk metallic glass has the capability to enhance the growth and specialization of osteoblasts in culture conditions.^[^ ^29^ ^]^ According to research, this material exhibits minimal degradation after a 4 weeks implantation period, yet it stimulates osteogenesis and the formation of new bone within two weeks of implantation.

By regulating the element composition and proportion of high‐entropy alloy, this material could be made more compatible with human tissues, reducing the rejection reaction and inflammatory reaction after implantation, facilitating cell adhesion and proliferation, and promoting the regeneration and repair of bone tissue.^[^ ^42^ ^]^ In addition, the light absorption of HEMXenes may promote the regeneration and repair of skin tissue while realizing antibacterial action (Figure 8G–I).^[^ ^43^ ^]^

In addition to directly using HEMs as the overall implant material, traditional implant material coatings composed of HEMs can improve the surface properties, biocompatibility, and corrosion resistance.^[^ ^41^ ^]^ HfNbTaTiZr high‐entropy alloy coatings can exhibit improved hardness, friction, and abrasion resistance, as well as biocompatibility after implantation.^[^ ^40^ ^]^ HEAs coatings can be deposited on metal substrates using different techniques, such as laser deposition, vapor deposition, and thermal spraying.^[^ ^105^ ^]^ Well‐designed HEMs, with excellent biocompatibility and unique element composition, as well as proper surface chemistry and customized surface topography, have gradually become outstanding candidates in the biomedical field.

As mentioned above, HEMs, as a bone implant material, is not only known for its excellent biological corrosion resistance and wear resistance, but also effectively alleviates the stress shielding effect through moderate yield strength, improving the overall fit of the implant. The incorporation of special elements gives HEMs the ability to enhance cell adhesion and growth, significantly improve biocompatibility, and reduce the risk of rejection and toxicity. In addition, the innovative design of high‐entropy materials, using the photothermal effect and ROS generation, shows the dual potential of antibacterial and promoting cell differentiation, opening up a new path for skin repair therapy. The combined effect of these characteristics makes HEMs show an unprecedented application prospect in the biomedical field.

## 6 Challenges and New Opportunities

Compared with traditional alloys, oxides, sulfide, and other compounds, HEMs offer an effective solution to the key challenges of functional nanomaterials in the biomedical field, mainly due to their high tunability and flexibility resulting from their multi‐dimensional component space. However, while some pioneering research results have been achieved, the following issues still need to be addressed.

1) Although the properties of HENMs can be customized through combination design, it remains a large challenge to develop more accurate and efficient synthesis methods and characterization techniques to obtain HENMs with higher structural controllability and better stability. We still need to explore the fine structure or morphological control (size, crystallinity, and morphology) of synthesized HENMs, as well as the important influence of composition and surface decoration on their performance.^[^ ^59b^ ^]^ Overall, research conducted remains in the early stages, and the modulation of properties appears to be conducted in a mostly random manner.

At present, it is extremely challenging to properly design HEMs. One possibility involves designing HEMs with the desired performance using the theoretical calculation method. Computational techniques (i.e., DFT calculations, machine learning) can be used to explore the change in functional properties under the intervention of various energy fields (i.e., light, sound, and electromagnetic fields) to screen HENMs candidate materials, to save on the time and cost of HENMs development. Another general strategy from an experimental point of view involves dividing components into sub‐categories to select a benchmark model according to their roles in the system, and then add or replace the selected elements in each category to change the configuration entropy of the system to obtain HENMs with unique performance. We expect that as our understanding of the synthesis–structure–performance relationship of HENMs continues to expand, the combination of machine learning optimization and screening will enable the discovery workflow of HEMs to accelerate progress in the biomedical field.

2) Research on the performance of biomedical high‐entropy materials (HENMs) is not comprehensive, and it remains challenging to explore new application scenarios. For example, HENMs containing heavy metals (i.e., W, Ta, Ga) have potential in CT/X‐ray imaging applications, while HENMs containing radioactive metals are suitable for PET imaging. In addition, the catalytic properties of HENMs are expected to be introduced into biological systems to catalyze specific biological reactions. By combining HENMs with other functional materials to form a hybrid material, the advantages of both sides can be combined, and performance superposition can be achieved, laying a foundation for multi‐functional biomedical applications. New preparation methods and surface modification technologies have been developed for specific needs to meet new demands in the biomedical field. To develop multi‐functional and intelligent high‐entropy nanomaterials to adapt to complex and dynamic biological environments, it is crucial to strengthen interdisciplinary cooperation and exchanges and promote the in‐depth research and innovative development of high‐entropy nanomaterials in the biomedical field.
3) The transport, transfer, digestion, absorption, and metabolism process of HENMs in vivo must be further studied to assess their biosafety and potential toxicity and achieve the more accurate positioning and controllable release of HENMs in vivo compared with other drug control materials. In addition, the immunogenicity and biological distribution of HEMs must be studied in large quantities. Therefore, it is critical to improve the biological performance and biocompatibility of HENMs through comprehensive biological evaluation and ultimately verify their safety and efficacy in clinical trials.
4) The long‐term biosafety and effectiveness of HENMs also require additional validation to evaluate their efficacy and safety at different stages to provide a reliable basis for clinical application. Even though research has demonstrated the general biocompatibility of HENMs currently employed in biomedical applications, a preponderance of these studies has been confined to cellular experiments or brief hematological assessments. Consequently, it is imperative to conduct comprehensive studies in the future to investigate the long‐term biosafety of HENMs, thereby paving the way for their expanded utilization in biomedical settings.

In summary, despite the above challenges, the results obtained thus far remain encouraging, and as technology continues to advance and HENMs continue to emerge, the application of HENMs in the biological field will become more extensive and in‐depth. Through collaborative research across various disciplines, we can expect to develop more efficient and safer biomedical HENMs to solve the above problems. In the future, HENMs will play a greater role in the field of biomedicine and provide greater contributions to human health.

## Conflict of Interest

The authors declare no conflict of interest.

## Author Contributions

All authors have read and approved the article. L.C. drafting and revise the important intellectual content of the manuscript; H.J. design part of the figure's; X.L. obtained funding; C.L., C.Q., and X.L. Supervised.
